# The Effect of Dietary Micelle Quercetin Supplementation on Growth Performance, Nutrient Digestibility, Gas Emission, Fecal Score, and Blood Profile in Growing‐Finishing Pigs

**DOI:** 10.1002/fsn3.72363

**Published:** 2026-10-21

**Authors:** Golam Sagir Ahammad, In Ho Kim

**Affiliations:** ^1^ Department of Animal Biotechnology Dankook University Cheonan Republic of Korea; ^2^ Smart Animal Bio Institute Dankook University Cheonan Republic of Korea

**Keywords:** bile acids, growth performance, micellar formulation, nutrient digestibility

## Abstract

This research explored the impact of dietary micelle quercetin (MQ) supplementation on growth metrics and health indicators in growing pigs. A total of 105 pigs (average body weight: 24.3 ± 3.51 kg) were randomly assigned to one of three dietary treatments for a 15 week trial. A control group (CON) received the basal diet, while TRT1 and TRT2 diets were supplemented with 250 mg MQ/kg and 500 mg MQ/kg, respectively. Each treatment had seven replicates, with five pigs per replicate. The study evaluated growth performance, nutrient digestibility, fecal consistency, noxious gas emissions, and blood biochemical parameters. Results showed that MQ supplementation had no significant effect on growth rates, feed utilization, fecal scores, or gaseous emissions. However, serum bile acid concentration was significantly influenced by MQ supplementation (*p* = 0.024), with the TRT2 group exhibiting higher levels than TRT1. In contrast, the control group (CON) did not differ significantly from either treatment. Meanwhile, liver enzyme activities (AST and ALT) remained unchanged (*p* > 0.05), suggesting no evidence of hepatic toxicity. In summary, MQ had no impact on overall performance and digestive traits but modulated bile acid profiles in a dose‐dependent manner, which may suggest a possible role in targeted metabolic modulation.

## Graphical abstract

Dietary micelle quercetin (MQ) supplementation at 250 or 500 mg/kg did not significantly affect growth performance, nutrient digestibility, fecal consistency, or noxious gas emissions in growing pigs. However, MQ supplementation significantly affected serum bile acid concentration (*p* = 0.024), with the 500 mg/kg MQ group showing higher levels than the 250 mg/kg group, while the control group did not differ significantly from either treatment. Serum AST and ALT activities remained unchanged, indicating no evidence of hepatic toxicity. Overall, MQ did not improve growth or digestive traits but may modulate bile acid metabolism in a dose‐dependent manner.

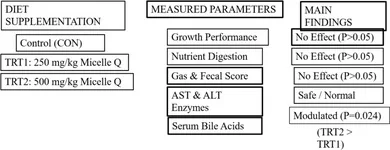

## 1 Introduction

In modern swine production, the demand for effective alternatives to antibiotic growth promoters has increased due to concerns over antimicrobial resistance and regulatory restrictions. Phytogenic compounds such as quercetin, a natural flavonoid widely found in fruits and vegetables (Anand David et al. 2016), have emerged as promising candidates. Quercetin exhibits strong antioxidant, anti‐inflammatory, and antimicrobial properties, which may help enhance gut health, immune function, and overall growth performance in pigs. Quercetin derived from *Sophora japonica* has been demonstrated to possess a promising effect in various animal models (Ahammad et al. 2024a; Ahammad and Kim 2024), but its application in livestock production, particularly for growing‐finishing pigs, remains underexplored.

Dietary supplementation with quercetin could influence various physiological and metabolic processes in pigs, potentially leading to improved growth performance and health (Park et al. 2020). However, one of the major challenges associated with quercetin use is its poor bioavailability, which limits its effectiveness in vivo (Anand David et al. 2016). To address this, micelle technology has been employed to enhance the absorption and efficacy of quercetin in the gastrointestinal tract (Dhanya 2022; Ahammad and Kim 2025a, 2025b, 2025c; Ahammad et al. 2024).

Previous studies have indicated that quercetin supplementation can positively impact growth performance and nutrient utilization in pigs. For example, piglets fed a basal diet supplemented with 400 mg/kg quercetin exhibited significantly higher final body weight and average daily gain compared to the control group (Mei et al. 2022). Additionally, Mao et al. (2024) reported that a dietary supplementation of 750 mg/kg quercetin improved feed conversion efficiency and decreased diarrhea incidence in weaned piglets by modulating gut microbiota composition and regulating the NF‐κB signaling pathway in the colon. However, the effects on other parameters such as gas emission, fecal characteristics, and blood profiles (including bile acid, AST, and ALT) are not well documented. These parameters are critical as they provide insights into the overall metabolic health, liver function, and environmental impact of pig farming.

We hypothesized that supplementing diets with micelle quercetin (MQ) may enhance growth and nutrient utilization while supporting normal blood profiles in growing‐finishing pigs. Therefore, this study aims to evaluate the effects of dietary micelle quercetin derived from *Sophora japonica* extract on the growth performance, nutrient digestibility, gas emission, fecal score, and blood profiles in growing‐finishing pigs.

## 2 Materials and Methods

Prior to initiating the feeding trial, the study protocol was carefully reviewed and approved by the Animal Care and Ethics Committee at Dankook University, South Korea. Ethical clearance for the research was granted under the approval number DK‐1‐2130, following a comprehensive assessment process.

### 2.1 Experimental Design, Animals, Housing and Diets

A 15‐week feeding experiment was conducted using 105 crossbred pigs (Landrace × Yorkshire × Duroc) with an average starting body weight of 24.3 ± 3.51 kg. The pigs were assigned to three dietary treatments following a randomized complete block design, stratified by sex (three gilts and two barrows per pen) and initial body weight to ensure uniformity. Each treatment group included seven pens, with five pigs per pen. Animals were housed in a temperature‐controlled facility and provided unrestricted access to feed and water. Diets were prepared in mash form and formulated according to the nutrient recommendations of the NRC (2014), delivered in three distinct feeding phases: grower (weeks 0–5), early finisher (weeks 6–10), and late finisher (weeks 11–15) (Table 1). The key dietary variable was quercetin (97% purity, Sigma‐Aldrich, USA), which was micellized using food‐grade emulsifiers to improve its bioavailability. Briefly, quercetin powder was dispersed in an aqueous solution containing a blend of food‐grade emulsifiers and surfactants, followed by high‐shear homogenization to form nanoscale micelles. To prepare a stable emulsion using Quercetin (97% purity, Sigma‐Aldrich, USA), researchers typically utilize a combination of non‐ionic surfactants and lipophilic emulsifiers to overcome the compound's practical insolubility in water. A common formulation protocol involves dissolving approximately 300 mg of quercetin into an oil phase, such as 3 g of Caprylic/capric triglyceride (Captex 355), which is then blended with 3 g of Tween 80 (Polysorbate 80) at 65°C for 1 h. This primary mixture is subsequently combined with an aqueous phase—often consisting of ultrapure water or a 10 mM Phosphate Buffer (pH 7.0) —supplemented with additional stabilizers like 1.5 g of soy lecithin and 15 g of 5% sodium alginate to enhance kinetic stability. The homogenization process is critical and is generally carried out using a high‐speed device like the Ultra‐Turrax T‐25 at 12,000 to 14,500 rpm for a duration of 4 min. Ultra‐pure (Milli‐Q) water as aqueous solution is used to prevent degradation, and the entire process should ideally be performed in an ice bath to maintain a temperature of 25°C, preventing thermal degradation of the flavonoid. The formation and optimization of the nanoscale micelles were confirmed through Dynamic Light Scattering (DLS) using a Zeta‐potential analyzer (e.g., Malvern Zetasizer), which is the gold standard for this scale. To arrive at the final formulation—yielding 20% micellar quercetin with 2% active quercetin—a series of surfactant‐to‐oil ratios were tested to minimize the Polydispersity Index (PDI), ensuring a uniform size distribution. The “nanoscale” nature was verified by a measured mean hydrodynamic diameter typically falling between 20 and 150 nm. Furthermore, Zeta potential analysis was employed to ensure physical stability; a value of approximately **−**20 mV to −30 mV (or higher) indicates strong electrostatic repulsion between micelles, preventing aggregation over time. To visually corroborate these results, Transmission Electron Microscopy (TEM) was likely used to observe the spherical morphology and actual particle size of the micelles, ensuring the quercetin was successfully encapsulated within the hydrophobic core of the surfactant matrix.

**TABLE 1:** Composition of growing pig diets.

Item	Growing	Finishing Phase 1	Finishing Phase 2
Ingredients (%)			
Corn	69.78	73.19	78.66
Soybean meal	19.34	16.75	11.52
Palm kernel meal	5.00	5.00	5.00
Tallow	2.54	2.21	2.06
Mono dicalcium phosphate	1.45	1.20	1.05
Limestone	0.75	0.64	0.62
Salt	0.20	0.10	0.10
Methionine (99%)	0.05	0.06	0.09
Lysine (78%)	0.46	0.42	0.47
Mineral mix^a^	0.20	0.20	0.20
Vitamin mix^b^	0.20	0.20	0.20
Choline (25%)	0.03	0.03	0.03
Total	100.00	100.00	100.00
Calculated value			
Crude protein, %	16.00	15.00	13.00
Metabolizable energy (kcal/kg)	3300	3300	3300
Calcium, %	0.70	0.60	0.55
Phosphorus, %	0.60	0.55	0.50
Lysin, %	1.10	1.00	0.90
Methionine, %	0.30	0.30	0.30
Fat, %	5.21	4.97	4.93

^a^ Provided per kilograms of diet: Fe, 100 mg as ferrous sulfate; Cu, 17 mg as copper sulfate; Mn, 17 mg as manganese oxide; Zn, 100 mg as zinc oxide; I, 0.5 mg as potassium iodide; and Se, 0.3 mg as sodium selenite.

^b^ Provided per kilograms of diet: vitamin A, 10,800 IU; vitamin D3, 4000 IU; vitamin E, 40 IU; vitamin K3, 4 mg; vitamin B1, 6 mg; vitamin B2, 12 mg; vitamin B6, 6 mg; vitamin B12, 0.05 mg; biotin, 0.2 mg; folic acid, 2 mg; niacin, 50 mg; D‐calcium pantothenate, 25 mg.

The resulting formulation contained at least 20% micellar quercetin and 2% active quercetin. Encapsulation efficacy was not directly measured in this study. The micellar quercetin formulation was standardized to yield approximately 20% micellar quercetin with 2% active quercetin. Throughout the study, environmental factors and resource allocation were carefully controlled to reduce external variability.

### 2.2 Sampling and Measurements

Body weight was measured at baseline and weeks 5, 11, and 15. Daily feed intake was recorded, this feed intake was used to measure average daily gain (ADG), average daily feed intake (ADFI), and gain to feed ratio (G: F).

To evaluate the digestibility of dry matter (DM), nitrogen (N), and gross energy (GE), chromium oxide (Cr_2_O_3_) was included in the experimental diets at 0.20% starting 7 days prior to fecal sample collection and continuously administered to the pigs. At the conclusion of week 15, fecal samples were obtained from one randomly selected barrow and one gilt per pen using rectal palpation. The collected samples were combined by pen and immediately stored at −20°C for later analysis. Before conducting chemical analysis, the samples were oven‐dried at 60°C for 72 h and finely ground to pass through a 1 mm mesh screen. Nitrogen (N) content was analyzed using a Kjeltec 2300 nitrogen analyzer (Foss Tecator AB, Hoeganaes, Sweden), whereas GE content was measured utilizing a Parr 6100 oxygen bomb calorimeter (Parr Instrument Co., Moline, Illinois, USA). The analysis for DM, N, and GE was conducted following the procedures outlined by AOAC (2000).

In the study, fresh fecal samples were collected from two randomly selected pigs per pen. From each sample, 300 g was allocated for gas emission analysis. The samples were transferred into 2.6‐l perforated plastic containers, each sealed with adhesive tape to maintain controlled conditions while allowing gas exchange. These containers were incubated at ambient room temperature to facilitate microbial fermentation. Gas emissions were quantified using a MultiRAE Lite gas detector (Model PGM‐6208, RAE Systems, USA), calibrated to measure concentrations of ammonia (NH_3_), hydrogen sulfide (H_2_S), methyl mercaptans, carbon dioxide (CO_2_), and acetic acid. For sampling, a small aperture was punctured through the adhesive seal of each container. A 100 mL air aliquot was aspirated from 2 cm above the fecal surface using a standardized protocol. Following sampling, the aperture was resealed to preserve internal conditions. After a 48 h incubation period, headspace gas measurements were repeated using the same methodology. Final gas concentrations were derived by averaging the two sequential measurements (initial and post‐48 h) from each container, ensuring precision and minimizing variability in the dataset.

To assess fecal consistency, samples were collected from five pigs per pen across all dietary treatments. Fecal scoring was conducted during weeks 1, 5, 10, and 15 of the trial, with evaluations performed twice daily at 8:00 and 20:00 h to account for potential diurnal variations. A standardized fecal consistency scoring system was applied as follows: 1 indicated firm, well‐formed stools; 2 indicated hard, dry pellets; 3 indicated soft, moist stools that maintained their shape; 4 indicated unformed, soft stools that took the shape of the container; and 5 indicated watery, liquid‐like stools.

At the end of the 15 weeks, seven pigs per treatment were randomly chosen (one pig per pen) and subjected to jugular venipuncture to collect blood samples. Approximately 5 mL blood samples were drawn from the selected pigs at different collection intervals into vacuum tubes devoid of additives to obtain serum. After centrifugation at 3000 × g for 15 min at 4°C, the serum was separated and stored at the same temperature until it underwent further analysis. The levels of AST, ALT, and bile acids in the serum samples were measured using an Automatic Biochemical Blood Analyzer (HITACHI 747, Tokyo, Japan).

### 2.3 Statistical Analysis

All collected data were analyzed using the Generalized Linear Model (GLM) procedure of SAS (SAS Institute Inc., Cary, NC, USA) under a randomized block design. The replicated pen served as the experimental unit. When the overall treatment effect was significant (*p* < 0.05), Duncan's multiple range test was applied as a post hoc procedure to separate means. Orthogonal polynomial contrasts were used to evaluate linear and quadratic responses to increasing dietary MQ concentrations; these effects were interpreted only when the overall model indicated significance. Trends were declared at *p* < 0.10.

## 3 Result and Discussion

In the present study, supplementation with MQ did not result in significant changes in body weight, ADG, ADFI, and G: F in growing‐finishing pigs (*p* > 0.05; Table 2). These findings contrast with previous research reporting growth‐promoting effects of quercetin in other animal models. For example, Abdel‐Latif et al. (2021) showed that dietary quercetin at 200, 400, and 800 ppm significantly enhanced growth performance in broiler chickens. Similarly, piglets receiving 400 mg/kg quercetin exhibited increased final body weight and ADG compared to controls (Mei et al. 2022), and Zou et al. (2016) observed improved ADG in pigs supplemented with 25 mg/kg quercetin. Positive effects have also been observed in rabbits, where supplementation with 2000 mg/kg quercetin dehydrate enhanced both growth and meat quality (North et al. 2019). The lack of response observed in the present study, despite the use of micellar quercetin, suggests that improved bioavailability alone may not be sufficient to elicit measurable improvements in growth performance under the experimental conditions. Growth responses to quercetin are likely influenced by multiple factors, including the physiological status of the animals, supplementation dose, duration of feeding, basal diet composition, and the biological activity of the quercetin formulation.

**TABLE 2:** The effect of dietary Micelle quercetin on growth performance in growing‐finishing pigs^a^.

Items	CON	TRT1	TRT2	SEM^b^	*p*	
					Linear	Quadratic
Body weight, kg						
Initial	23.33	23.30	23.30	0.01	0.728	0.840
Week 5	46.51	46.76	47.02	0.34	0.253	0.934
Week 10	75.48	76.34	76.91	0.75	0.228	0.890
Finish	106.55	108.18	108.88	1.21	0.277	0.833
Initial—Week 5						
ADG, g	658	670	678	10	0.255	0.929
ADFI, g	1515	1525	1529	13	0.531	0.860
G: F	0.434	0.439	0.443	0.007	0.528	0.994
Week 5–10						
ADG, g	831	845	854	12	0.232	0.859
ADFI, g	2519	2531	2537	28	0.674	0.919
G: F	0.329	0.333	0.336	0.007	0.554	0.940
Week 10–15						
ADG, g	894	910	913	13	0.385	0.755
ADFI, g	2830	2840	2847	38	0.799	0.987
G: F	0.316	0.320	0.321	0.009	0.758	0.867
Overall						
ADG, g	795	808	815	12	0.283	0.839
ADFI, g	2288	2299	2304	20	0.634	0.931
G: F	0.347	0.351	0.354	0.008	0.608	0.935

*Note: n* = 7 pens/treatment. Power sufficient for moderate effects; small effects may remain undetected.

Abbreviations: ADG, average daily gain; ADFI, average daily feed intake; G:F, gain to feed ratio.

^a^ CON, Basal diet; TRT1, Basal diet + 250 mg/kg Micelle Quercetin; TRT2, Basal diet + 500 mg/kg Micelle Quercetin.

^b^ Standard error of means.

Dietary fat may also contribute to the effectiveness of quercetin because lipid intake has been reported to enhance the absorption of conventional quercetin (Majid et al. 2024). A study by Lesser et al. (2004) demonstrated that quercetin bioavailability was significantly greater when diets contained 17% fat compared to only 3%. However, the present study did not measure quercetin bioavailability, and therefore it cannot be concluded that the relatively low dietary fat content was the primary reason for the absence of growth responses. Furthermore, dietary fat levels in Mei et al. (2022) and Zou et al. (2016) were followed by standard swine feeding practices, suggesting that differences in ADG among studies are more likely related to quercetin form, dose, or experimental conditions rather than fat content. Similarly, although Park et al. (2020) reported dietary fat levels comparable to ours, improved ADG during the first 21 days may reflect the use of free quercetin.

Overall, the discrepancies among studies are likely attributable to multiple factors, including differences in quercetin formulation, supplementation dosage, animal physiological status, and experimental conditions, rather than dietary fat content alone. Although micellar technology has the potential to enhance quercetin delivery and bioavailability, improved absorption does not necessarily result in enhanced growth performance. Therefore, further research is warranted to elucidate the relationship between quercetin bioavailability, pharmacokinetics, and physiological responses under varying dietary and production conditions.

In this study, dietary supplementation with MQ did not significantly affect the apparent total tract digestibility of nutrients (*p* > 0.05; Table 3). This finding contrasts with some prior studies that reported improved nutrient utilization following flavonoid supplementation. For instance, Dang et al. (2022) observed enhanced dry matter digestibility and energy retention in broilers supplemented with quercetin at 200–600 mg/kg, suggesting a potential dose‐ or species‐specific effect. Similarly, Wang et al. (2007) reported improved digestibility in pigs fed herbal additives, likely through modulation of gut function. However, our results align with other studies reporting non‐significant impacts of flavonoids or phytogenic additives on nutrient digestibility. Castillo et al. (2006), Cui et al. (2019), and Ao et al. (2011) each observed no notable improvements in digestibility in pigs supplemented with various plant‐based compounds. One possible explanation is that certain dietary phytochemicals, such as capsaicin and piperine, can modulate enteric neural signaling and smooth muscle activity, thereby enhancing gastrointestinal motility and accelerating intestinal transit, which may consequently limit nutrient absorption (Debreceni et al. 1999; Gonzalez et al. 1998; Mózsik et al. 2007). Additionally, Hossain et al. (2024) reported that a low dose of quercetin had no significant effect on nutrient digestibility in pigs. Therefore, in our study, the lack of effect of micellar quercetin on nutrient digestibility may be due to low dosage and potential modulation of gut motility, which could accelerate intestinal transit and limit nutrient absorption.

**TABLE 3:** The effect of dietary Micelle Quercetin on nutrient digestibility in growing—finishing pigs^a^.

Items, %	CON	TRT1	TRT2	SEM^b^	*p*	
					Linear	Quadratic
Finish						
Dry matter	70.42	70.70	71.01	0.91	0.677	0.999
Nitrogen	68.55	68.57	68.81	0.87	0.858	0.926
Digestible energy	69.42	69.82	69.93	0.89	0.688	0.886

*Note: n* = 7 pens/treatment. Power sufficient for moderate effects; small effects may remain undetected.

^a^ CON, Basal diet; TRT1, Basal diet + 250 mg/kg Micelle Quercetin; TRT2, Basal diet + 500 mg/kg Micelle Quercetin.

^b^ Standard error of means.

Micellar quercetin (MQ) supplementation did not significantly affect the emission of ammonia (NH_3_), hydrogen sulfide (H_2_S), or other measured gases from pig manure (*p* > 0.05; Table 4). Given that gas emissions are closely linked to nutrient digestibility and microbial fermentation in the gut, the absence of digestibility improvement in our study likely contributed to the unchanged gas output. While some studies have shown that flavonoid‐rich diets can modulate gut microbiota and reduce gas production—primarily in poultry (Jeong and Kim 2014)—others have reported no significant effects. For example, Jiao et al. (2019) found that flavonoid supplementation did not alter gas emissions or cecal microbial composition in broilers. These inconsistencies may be due to differences in flavonoid type, dosage, animal species, and experimental conditions. In our case, the dose of MQ used may have been too low to alter the microbial community and thereby affect gas production. However, this study's design did not include microbial community analysis to verify this mechanism. A key limitation is that we did not analyze the microbial community. Further studies are needed to clarify the mechanisms by which dietary flavonoids may influence fermentation profiles and gaseous emissions in swine.

**TABLE 4:** The effect of dietary Micelle Quercetin on gas emission in growing—finishing pigs^a^.

Items, ppm	CON	TRT1	TRT2	SEM^b^	*p*	
					Linear	Quadratic
Finish						
NH_3_	6.98	6.88	6.63	0.50	0.719	0.945
H_2_S	5.58	5.28	5.05	0.51	0.469	0.938
Methyl mercaptans	4.73	4.38	4.00	0.58	0.404	1.000
Acetic acid	12.49	12.38	11.75	0.55	0.252	0.643
CO_2_	15,374	15,075	14,975	543	0.541	0.858

*Note: n* = 7 pens/treatment. Power sufficient for moderate effects; small effects may remain undetected.

^a^ CON, Basal diet; TRT1, Basal diet + 250 mg/kg Micelle Quercetin; TRT2, Basal diet + 500 mg/kg Micelle Quercetin.

^b^ Standard error of means.

Fecal consistency, as assessed by fecal scores, was not significantly affected by MQ supplementation (*p* > 0.05; Table 5). As fecal scores are commonly used to evaluate gastrointestinal health, particularly the presence of diarrhea, the lack of changes suggests that MQ did not impair intestinal function or tolerance. These results are consistent with previous studies reporting no significant effects of certain phytogenic additives on fecal consistency in pigs (Zhang et al. 2012, 2023). In contrast, other reports have indicated that flavonoids may exert prebiotic effects by modulating gut microbiota, potentially reducing diarrhea in early post‐weaning pigs (Haghshenas et al. 2023). However, since our study did not include microbial profiling, no conclusions can be drawn regarding potential microbiota shifts. Overall, the results indicate that MQ was at least neutral in terms of digestive health, and further studies incorporating microbiome analyses are warranted to explore its broader effects on gut function.

**TABLE 5:** The effect of dietary Micelle Quercetin on fecal score in growing—finishing pigs^a^.

Items	CON	TRT1	TRT2	SEM^b^	*p*	
					Linear	Quadratic
Fecal score^c^						
Week 1	3.25	3.25	3.25	0.05	0.912	0.909
Week 5	3.26	3.25	3.24	0.04	0.775	0.840
Week 10	3.23	3.21	3.20	0.03	0.688	0.938
Week 15	3.18	3.17	3.15	0.04	0.781	0.719

*Note: n* = 7 pens/treatment. Power sufficient for moderate effects; small effects may remain undetected.

^a^ CON, Basal diet; TRT1, Basal diet + 250 mg/kg Micelle Quercetin; TRT2, Basal diet + 500 mg/kg Micelle Quercetin.

^b^ Standard error of means.

^c^ Fecal score = 1 hard, dry pellet; 2 firm, formed stool; 3 soft, moist stool that retains shape; 4 soft, unformed stool that assumes shape of container; 5 watery liquid that can be poured.

Serum bile acid concentration was significantly influenced by MQ supplementation (*p* = 0.024). The TRT2 group (500 mg/kg) exhibited significantly higher levels compared to TRT1 (250 mg/kg), whereas the control group (CON) showed no significant difference relative to either TRT1 or TRT2. In contrast, serum liver enzymes AST and ALT remained unchanged (*p* > 0.05; Table 6), indicating no signs of liver toxicity within the trial period. Serum ALT (31–58 U/L) and AST (32–84 U/L) activities remained within the normal physiological ranges reported for healthy pigs, supporting the absence of hepatic toxicity (Hakze‐Van Der Honing et al. 2011). The rise in bile acid concentrations suggests a dose‐dependent effect of quercetin on hepatic metabolism. Flavonoids like quercetin are known to interact with nuclear receptors such as farnesoid X receptor (FXR), which regulates bile acid synthesis and cholesterol homeostasis (Chiang and Fiorucci 2021; Liu et al. 2018). While this pathway may provide a possible explanation for the observed pattern, the current study did not directly assess FXR activity. Therefore, the modulation of bile acid metabolism through FXR signaling should be interpreted cautiously. Future transcriptomic or proteomic analyses would be valuable to clarify whether MQ supplementation alters FXR‐mediated pathways or associated bile acid transport mechanisms.

**TABLE 6:** The effect of dietary Micelle Quercetin on blood profile in growing—finishing pigs*.

Items	CON	TRT1	TRT2	SEM**	Treatment (*p*)	Poly. contrast	
						Linear	Quadratic
Finish							
Bile acid, μmol/L	26.37^ab^	22.72^b^	29.25^a^	2.00	0.024	0.320	0.048
AST, U/L	55.6	54.7	61.8	3.6	0.352	0.222	0.369
ALT, U/L	48.6	50.0	47.3	3.1	0.657	0.789	0.583

*Note:* *n* = 7 pens/treatment. Power sufficient for moderate effects; small effects may remain undetected. ^a,b^Means in the same row with different superscripts differ significantly (*p* < 0.05).

Abbreviations: ALT, alanine aminotransferase; AST, aspartate aminotransferase.

* CON, Basal diet; TRT1, Basal diet +250 mg/kg Micelle Quercetin; TRT2, Basal diet +500 mg/kg Micelle Quercetin.

** Standard error of means.

Although liver enzyme levels did not indicate acute hepatic stress, sustained elevations in bile acids could potentially affect liver function over time. This aligns with findings that excessive bile acid accumulation can contribute to oxidative stress and inflammation in hepatic tissues (Chiang 2017). The micellar formulation likely improved quercetin's bioavailability compared with its conventional form, enabling greater metabolic activity within a safe range (Boots et al. 2008; Galati et al. 2002; Ahammad et al. 2024b) and possibly exceeding the optimal physiological threshold in TRT2. These results underscore the importance of dose consideration in MQ supplementation. While moderate levels may benefit lipid metabolism (Uylaş et al. 2018), higher doses might disrupt bile acid homeostasis.

### 3.1 Limitation of the Study

One limitation of the present study is that the dietary fat content (approximately 4.9%–5.2%), although representative of practical grower–finisher pig production, may not have been sufficient to maximize the absorption and physiological efficacy of micellar quercetin, given its known fat‐dependent bioavailability. Additionally, while a significant treatment effect on serum bile acid concentration was observed, the response was modest and not consistently different from the control group, limiting the strength of biological interpretation. Furthermore, the study did not include direct measurements of quercetin bioavailability or molecular markers (e.g., FXR signaling), which restricts mechanistic insight into the observed effects. A further limitation is that complete sex balance across treatments was not strictly controlled and is acknowledged as a limitation of the study.

## 4 Conclusion

In this study, dietary supplementation with MQ derived from *Sophora japonica* at levels of 250 mg/kg and 500 mg/kg did not significantly influence growth performance, nutrient digestibility, fecal consistency, or gaseous emissions in growing–finishing pigs. However, serum bile acid concentrations exhibited a quadratic response, while serum AST and ALT activities remained unchanged across treatments. These findings suggest that MQ may affect hepatic metabolism without causing liver toxicity. Further studies are recommended to clarify the metabolic role of MQ, enhance its bioavailability, and investigate its impact on liver function and related molecular pathways.

## Author Contributions

**Golam Sagir Ahammad:** conceptualization, investigation, writing – original draft, methodology, software, formal analysis, project administration, data curation, supervision, visualization, writing – review and editing. **In Ho Kim:** investigation, funding acquisition, writing – review and editing, validation, project administration, supervision.

## Funding

This research was supported by the Basic Science Research Programmed through the National Research Foundation of Korea (NRF) funded by the Ministry of Education (NRF‐RS‐2023‐00275307).

## Ethics Statement

All procedures were approved by Animal Care and Committee of Dankook University. The procedures were set to fulfill the requirements of European Union Legislation (2010/63/EU Directive), Official Journal of the European Union, Luxembourg.

## Conflicts of Interest

The authors declare no conflicts of interest.

## Data Availability

The data that support the findings of this study are available from the corresponding author upon reasonable request.
